# Tracking the evolution of anti-SARS-CoV-2 antibodies and long-term humoral immunity within 2 years after COVID-19 infection

**DOI:** 10.1038/s41598-024-64414-9

**Published:** 2024-06-11

**Authors:** Mariam Movsisyan, Nune Truzyan, Irina Kasparova, Armine Chopikyan, Ra’ed Sawaqed, Alexandra Bedross, Meline Sukiasyan, Karen Dilbaryan, Sanobar Shariff, Burhan Kantawala, Gohar Hakobjanyan, Gayane Petrosyan, Armine Hakobyan, Konstantin Yenkoyan

**Affiliations:** 1 Department of Allergology and Clinical Immunology, Yerevan State Medical University Named After Mkhitar Heratsi, Yerevan, Armenia; 2 Cobrain Center, Yerevan State Medical University Named After Mkhitar Heratsi, Yerevan, Armenia; 3 Department of Histology, Yerevan State Medical University Named After Mkhitar Heratsi, Yerevan, Armenia; 4 Department of Public Health and Healthcare Organization, Yerevan State Medical University Named After Mkhitar Heratsi, Yerevan, Armenia; 5 General Medicine Faculty, Yerevan State Medical University Named After Mkhitar Heratsi, Yerevan, Armenia; 6 Laboratory-Diagnostic Center of Heratsi Clinical Hospital, Yerevan State Medical University Named After Mkhitar Heratsi, Yerevan, Armenia; 7 Neuroscience Laboratory, Cobrain Center, Yerevan State Medical University Named After Mkhitar Heratsi, 0025 Yerevan, Armenia; 8 Department of Biochemistry, Yerevan State Medical University Named After Mkhitar Heratsi, Yerevan, Armenia

**Keywords:** Anti-SARS-CoV-2 (S), Anti-SARS-CoV-2 (N), Kinetics, Long-term humoral immunity, Biochemistry, Immunology, Diseases

## Abstract

The severe acute respiratory syndrome coronavirus 2 (SARS-CoV-2) that gave rise to COVID-19 infection produced a worldwide health crisis. The virus can cause a serious or even fatal disease. Comprehending the complex immunological responses triggered by SARS-CoV-2 infection is essential for identifying pivotal elements that shape the course of the disease and its enduring effects on immunity. The span and potency of antibody responses provide valuable perspicuity into the resilience of post-infection immunity. The analysis of existing literature reveals a diverse controversy, confining varying data about the persistence of particular antibodies as well as the multifaceted factors that impact their development and titer, Within this study we aimed to understand the dynamics of anti-SARS-CoV-2 antibodies against nucleocapsid (anti-SARS-CoV-2 (N)) and spike (anti-SARS-CoV-2 (N)) proteins in long-term immunity in convalescent patients, as well as the factors influencing the production and kinetics of those antibodies. We collected 6115 serum samples from 1611 convalescent patients at different post-infection intervals up to 21 months Study showed that in the fourth month, the anti-SARS-CoV-2 (N) exhibited their peak mean value, demonstrating a 79% increase compared to the initial month. Over the subsequent eight months, the peak value experienced a modest decline, maintaining a relatively elevated level by the end of study. Conversely, anti-SARS-CoV-2 (S) exhibited a consistent increase at each three-month interval over the 15-month period, culminating in a statistically significant peak mean value at the study’s conclusion. Our findings demonstrate evidence of sustained seropositivity rates for both anti-SARS-CoV-2 (N) and (S), as well as distinct dynamics in the long-term antibody responses, with anti-SARS-CoV-2 (N) levels displaying remarkable persistence and anti-SARS-CoV-2 (S) antibodies exhibiting a progressive incline.

## Introduction

The severe acute respiratory syndrome coronavirus 2 (SARS-CoV-2) that gave rise to COVID-19 infection produced a worldwide health crisis^1–3^. SARS-CoV-2, a positive-sense single-stranded RNA virus, possesses an array of structural and non-structural proteins that include the envelope, membrane protein, spike protein, and nucleocapsid protein, with a viral entry into the human cells facilitated by binding of spike protein to the ACE2 receptor on the cell surface^7–9^ .

The virus can cause a serious or even fatal disease, especially in those with underlying medical disorders^4–6^. Comprehending the complex immunological responses triggered by SARS-CoV-2 infection is essential for identifying pivotal elements that shape the course of the disease and its enduring effects on immunity. Producing antibodies against spike protein (anti-SARS-CoV-2 (S)) after infection or following vaccination is the natural humoral immune response, that is responsible for enduring long-term protective immunity. In contrast, the nucleocapsid protein inhabiting the virus is crucial for preserving its genetic material and necessary for replication and grouping. Various studies indicate a reduced effectiveness of antibodies against nucleocapsid protein (anti-SARS-CoV-2 (N)) in neutralizing the virus^10^, thereby illustrating the importance of a better comprehension of immune responses in the context of COVID-19 infection.

The span and potency of antibody responses provide valuable perspicuity into the resilience of post-infection immunity. The analysis of existing literature reveals a diverse controversy, confining varying data about the persistence of particular antibodies as well as the multifaceted factors that impact their development and titer, notably, anti-SARS-CoV-2 (N) demonstrated reduced persistence in comparison to anti-SARS-CoV-2 (S), especially within the initial year following infection with SARS-CoV-2. Due to markedly elevated peak levels of anti-SARS-CoV-2 (S), the state of anti-SARS-CoV-2 (S) seropositivity persisted for over 14 months, in stark contrast to the period of less than one year observed for anti-SARS-CoV-2 (N) in individuals exhibiting both non-severe and severe manifestations of COVID-19 infection^11^. In most cases, levels of anti-SARS-CoV-2 (N) reached their peak around 90–100 days post-infection^12^. Studies revealed that anti-SARS-CoV-2 (S), are essential players in neutralizing the virus, and exhibit more persistence when compared to anti-SARS-CoV-2 (N)^13,14^.

While the number of publications studying humoral immunity following COVID-19 infection is plentiful, the number of studies evaluating the long-term humoral response after 15 months is limited.

A group of factors that impact on the dynamics of immune responses are age, gender, blood group, various comorbidities etc. Age, for instance, is a pivotal factor, and according to various studies, humoral immune responses wane in older individuals^15–17^. Higher antibody levels are associated with increased disease severity and the risk of severe COVID-19 infection is connected to characteristics such as age, male sex, and various comorbidities, including the immunocompromised condition^18–20^.

Beyond age, gender also plays a role, as females have been shown to exhibit stronger inflammatory, antiviral, and humoral immune responses during viral infections^21–23^. It is noteworthy that individuals with severe COVID-19 disease exhibited higher levels of anti-SARS-CoV-2 (S) antibodies relative to those with non-severe disease^24–26^.

The Rh factor also influences the severity of the COVID-19 infection. The Rh− blood group is linked to a decreased probability of acquiring severe COVID-19 infection, implying that the Rh− blood group may protect against severe SARS-CoV-2 disease. Blood types A, B, and Rh+ are reported to be more vulnerable to COVID-19 infection, whereas blood groups O, AB, and Rh− are less sensitive ^27–29^**.**

Analyzing the current literature can allow finding different gaps for a thorough comprehension of humoral immunity, which is a necessity in a way to develop effective evaluation tools for herd immunity, improved strategies for treatment, and vaccination protocols to decrease the mortality rate.

This investigation aims to comprehend the sophisticated dynamics of anti-SARS-CoV-2 (N) and (S) in long-term immunity in convalescent patients and glimpse factors influencing that. Tackling the intricacies of antibody response influenced by diverse factors enables the development of educated strategies to combat the virus and create effective treatments and preventative measures.

## Results

### General characteristics

A comprehensive cohort of 1611 laboratory-confirmed COVID-19 infection patients formed the subject of investigation in this study, to assess the antibody response dynamics after the disease. The demographic data of those patients were described in Table 1 regarding age, gender, disease severity, and comorbidities. The three most common concomitant disorders in the target group comprised cardiovascular diseases (281 patients), diabetes (77 patients), and autoimmune thyroiditis (60 patients). The allocation of participants according to the severity of COVID-19 infection was categorized into four groups based on the latest severity guidelines outlined by the World Health Organization (WHO) of which 921 individuals were within the severe group, 524 individuals—in the moderate group, 611 the mild group, and 55 individuals within the asymptomatic group. The composition of the cohort demonstrated a predominance of females, accounting for 73.2% of the total, and exhibited a mean age of 49.3 years, spanning an age range from 18 to 83 years (Table 1). Remarkably, the patients identified within the severe group exhibited a statistically significant advancement in age (mean 53.67 ± 13.97) compared to those belonging to the mild and asymptomatic categories (mean 43.48 ± 14.54). Among the 642 patients who developed pneumonia, 56.5% (n = 363) exhibited pulmonary involvement with a percent mean equal to 20.1 ± 15.0 (ranging from 5 to 90%) and 27.6% (n = 177) reported having saturation (SaO2) deficiency ranging from 73 to 95. The time interval between the initial PCR test report (baseline) for COVID-19 infection convalescent patients and their participation in this study (follow-up) varied, extending from a few days to 2 years, with a mean duration of 21.0 ± 14.0 weeks. The mean time between samplings and the average number of samples per subjects during our study are detailed in Supplementary Table S1. 61.94% of the study participants were sampled up to 3 times, 16.87% were sampled 4 to 6 times, while 21.19% were sampled 7 or more times.

**Table 1:** Demographic, behavioral, and clinical characteristics of patients diagnosed and followed-up for COVID-19 infection from August 2020 to March 2022 in Armenia.

	Total sample	Asymptomatic + Mild	Moderate + Severe	*P* value
Gender, n (%)				
Male	432 (26.9%)	256 (26.1%)	176 (28.3%)	> 0.05
Female	1170 (73.2%)	723 (73.9%)	447 (71.7%)	> 0.05
Age, mean, SE	47.7 (0.39)	43.6 (0.48)	53.6 (0.57)	< 0.05
BMI, n (%)				< 0.05
Underweight	43 (3.8%)	32 (4.7%)	11 (2.4%)	
Normal	436 (38.2%)	287 (41.8%)	149 (32.7%)	
Overweight	371 (32.5%)	221 (32.2%)	150 (33%)	
Obese	292 (25.6%)	147 (21.4%)	145 (31.9%)	
Blood group, n (%)				> 0.05
O	232 (26.5%)	137 (26.6%)	95 (26.2%)	
A	516 (59%)	308 (59.9%)	208 (57.5%)	
B	77 (8.8%)	45 (8.9%)	32 (8.8%)	
AB	50 (5.7%)	24 (4.7%)	26 (7.5%)	
Rhesus, n (%)				> 0.05
Negative	187 (20.1%)	108 (19.8%)	79 (20.5%)	
Positive	744 (79.9%)	437 (80.2%)	307 (79.5%)	
Cardiovascular diseases, n (%)	281 (18.5%)	140 (15.4%)	141 (23.3%)	< 0.05
Diabetes melitus, n (%)	77 (5.1%)	30 (3.3%)	47 (7.8%)	< 0.05
COPD, n (%)	4 (0.3%)	2 (0.2%)	2 (0.3%)	> 0.05
Asthma, n (%)	16 (1.1%)	9 (1%)	7 (1.2%)	> 0.05 ara>
Autoimmune Thyroiditis, n (%)	60 (4%)	36 (4%)	24 (4%)	> 0.05
Symptoms reported during Covid-19 infection, n (%)				
Temperature (N = 1508)	1262 (83.7%)	733 (81.4%)	529 (87.2%)	< 0.05
Cough (N = 1507)	517 (34.3%)	293 (32.4%)	224 (36.9%)	> 0.05
Shortness of breath (N = 1508)	415 (27.5%)	230 (25.4%)	185 (30.5%)	< 0.05
Weakness (N = 1508)	1081 (71.7%)	613 (68%)	468 (77.2%)	< 0.05
Body pain (N = 1509)	734 (48.6%)	419 (46.3%)	315 (52%)	< 0.05
Backpain (N = 1507)	363 (24%)	199 (22%)	164 (26.9%)	< 0.05
Diarrhea (N = 1500)	148 (9.8%)	71 (7.9%)	77 (12.6%)	< 0.05
Nausea (N = 1505)	175 (11.6%)	88 (9.8%)	87 (14.3%)	< 0.05
Vomiting (N = 1502)	73 (4.8%)	46 (5.1%)	27 (4.4%)	> 0.05
Headache (N = 1502)	748 (49.8%)	429 (47.8%)	319 (52.7%)	> 0.05
Dizziness (N = 1504)	503 (33.4%)	292 (32.5%)	211 (34.8%)	> 0.05
Muscle pain (N = 1507)	700 (46.4%)	400 (44.4%)	300 (49.2%)	> 0.05
Joint pain (N = 1505)	428 (28.4%)	245 (27.2%)	183 (30.2%)	> 0.05
Disorientation (N = 1504)	73 (4.9%)	42 (4.7%)	31 (5.1%)	> 0.05
Memory loss/disturbance (N = 1506)	139 (9.2%)	74 (8.2%)	65 (10.7%)	> 0.05
Sleep disturbance (N = 1503)	216 (14.4%)	116 (12.9%)	100 (16.4%)	> 0.05
Fear (N = 1500)	127 (8.5%)	66 (7.4%)	61 (10%)	> 0.05
Anxiety (N = 1504)	103 (6.8%)	52 (5.8%)	51 (8.4%)	< 0.05
Depressive symptoms (N = 1505)	134 (8.9%)	74 (8.2%)	60 (9.8%)	> 0.05
Smell/taste disturbance (N = 1506)	679 (45.2%)	390 (43.4%)	289 (47.8%)	> 0.05
Smell/taste loss (N = 1508)	595 (39.6%)	331 (36.8%)	264 (43.6%)	< 0.05
Visual disorder/diplopia (N = 1504)	50 (3.3%)	29 (3.2%)	21 (3.4%)	> 0.05
Speech disturbance (N = 1503)	37 (2.5%)	16 (1.8%)	21 (3.5%)	< 0.05
Swallow disturbance (N = 1506)	31 (2.1%)	16 (1.8%)	15 (2.5%)	> 0.05
Weakness of limbs (N = 1506)	280 (18.5%)	157 (17.4%)	123 (20.2%)	> 0.05
Numbness of face and/or limbs (N = 1508)	131 (8.7%)	67 (7.4%)	64 (10.7%)	< 0.05
Urinary disorder (N = 1507)	26 (1.7%)	9 (1%)	17 (2.8%)	< 0.05
Gait disturbance (N = 1503)	40 (2.7%)	16 (1.8%)	24 (3.9%)	< 0.05
Non-traumatic bruises (N = 1493)	22 (1.5%)	15 (1.7%)	7 (1.2%)	> 0.05

### Seroprevalence and kinetics of anti-SARS-CoV-2 (N) and (S) antibodies

Measurements of anti-SARS-CoV-2 (N) and anti-SARS-CoV-2 (S) showed that the level of antibodies and the dynamics extensively varied among COVID-19 convalescent patients. Supplementary Figure S1 illustrates the time-related change of anti-SARS-CoV-2 (N) and anti-SARS-CoV-2 (S) over 18 months, displaying the dynamic variations of 22 individuals throughout the follow-up period. These 22 patients were randomly selected from the subset of individuals who underwent a higher frequency of samplings.

**Figure 1:**
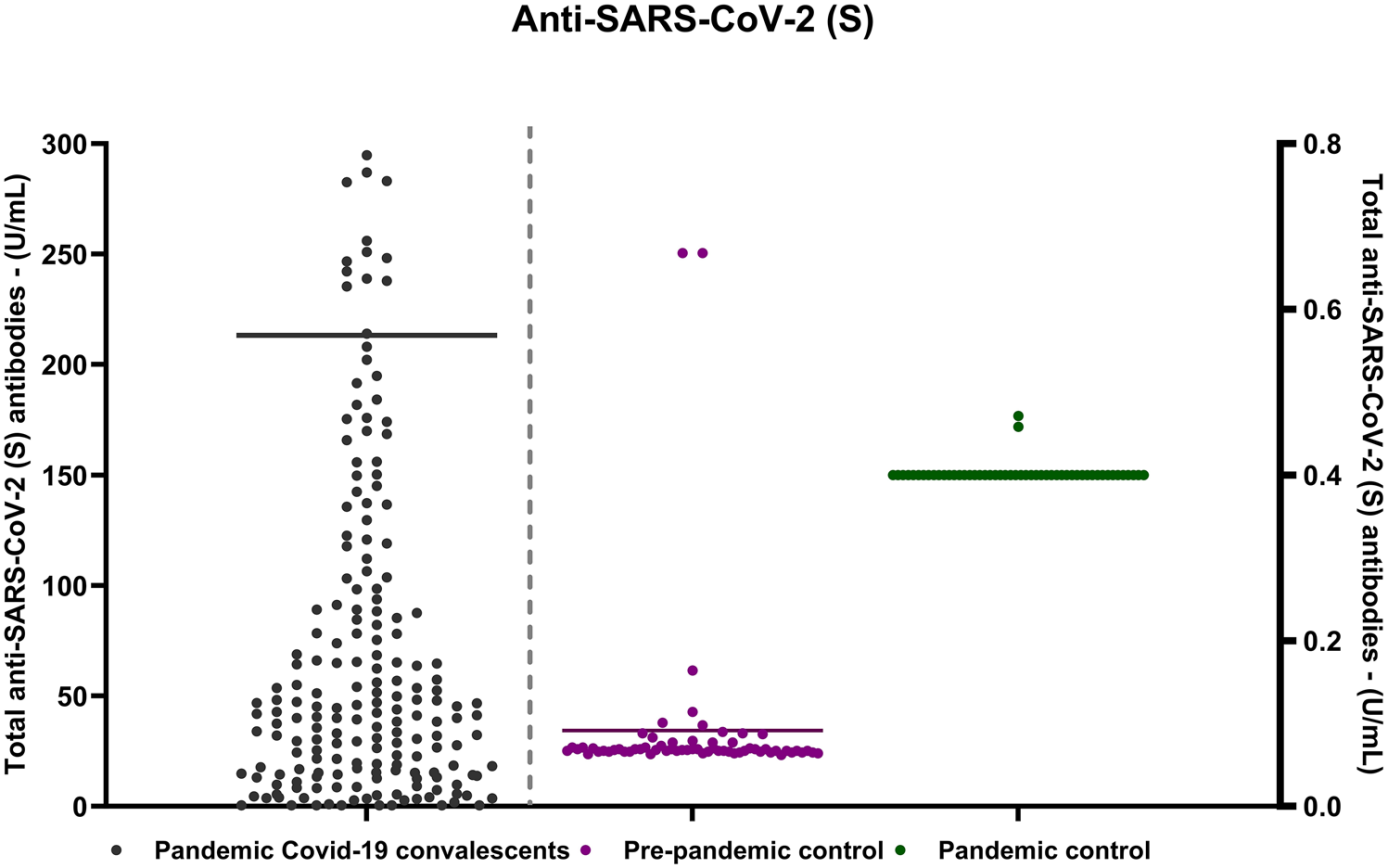
The scatter plot visually illustrates all observed samples, operating two y-axes with different measurement ranges. The experimental group axis, with a peak value of 300 U/ml, encompasses serum samples from 1661 patients. The control group axis has a peak value of 0.8 U/ml, representing the “pre-pandemic control group” including serum samples from healthy donors before and during the early phases of the SARS-CoV-2 pandemic. Values below 0.8 U/ml are considered negative, while those greater than or equal to 0.8 U/ml are deemed positive.

In this comprehensive study conducted over 21 months, data reveals intriguing insights into the antibody levels among the participants. Notably, 94.5% (n = 1521) of the subjects exhibited at least one positive value for anti-SARS-CoV-2 (N), while a slightly higher percentage, 95.9% (n = 1506), demonstrated positive anti-SARS-CoV-2 (S). Introducing an additional layer of intricacy to our observations is the subgroup of 45 patients displaying a distinctive pattern in their antibody response. In 1% of individuals within this subset exhibited positive anti-SARS-CoV-2 (S) alongside a simultaneous absence of anti-SARS-CoV-2 (N). In our previous study Movsisyan et al. (2022), we demonstrated that the anti-SARS-CoV-2 (N) levels in convalescent patients were significantly high compared to both pre-pandemic and pandemic healthy controls where anti-SARS-CoV-2 (N) was undetectable. Our current research reaffirms this pattern, indicating a similar trend for anti-SARS-CoV-2 (S) levels (Fig. 1).

### Kinetics and magnitude of anti-SARS-CoV-2 (N) and anti-SARS-CoV-2 (S) antibodies

**Figure 2:**
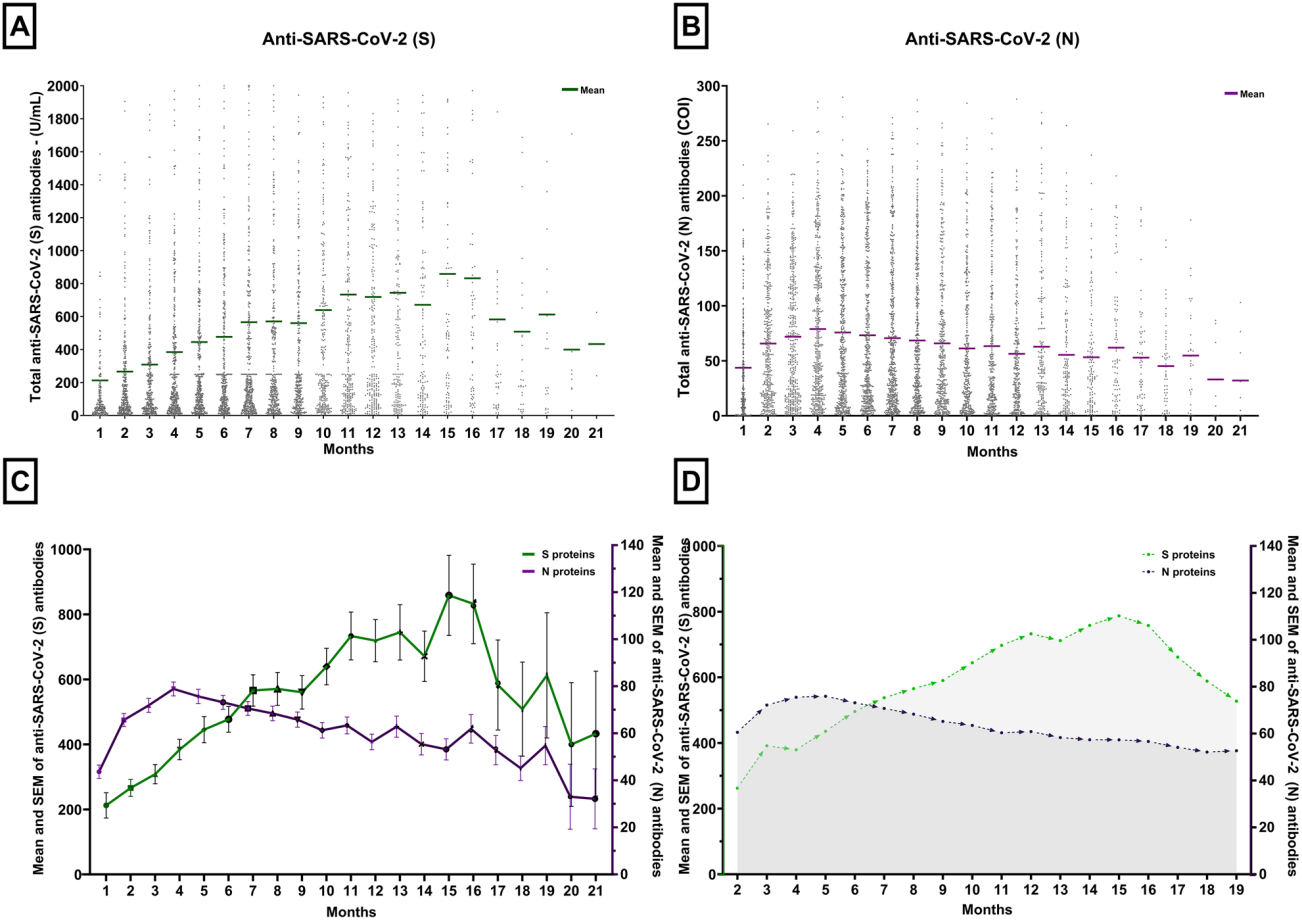
Seroprevalence of anti-SARS-CoV-2 (S) and (N). Figures (**A**) and (**B**) demonstrate anti-SARS-CoV-2 (S) and (N) antibodies of different convalescent patients with a 21-month follow-up and mean of each month. Figure C demonstrates the dynamics of anti-SARS-CoV-2 (S) and (N) within 21 months using two y-axes with different measurement ranges. The peak value for anti-SARS-CoV-2 (S) was 1000U/ml, for anti-SARS-CoV-2 (N)-140 COI.

The concentrations of anti-SARS-CoV-2 (N) and anti-SARS-CoV-2 (S) in the serum of tested patients increased gradually with occasional oscillations, beginning with the initial three-month findings (Fig. 2). Although the values recorded at 16–21 months of follow-up were eliminated from the statistical analysis due to sample size limits, both anti-SARS-CoV-2 (N) and anti-SARS-CoV-2 (S) were detectable until the end of the 21 months (Fig. 2A and B).

Standard error of mean for each month was exhibited. Figure **D** represents the moving average of anti-SARS-CoV-2 (S) and (N). To depict the temporal patterns of these changes, a moving average was calculated as the average of the current, previous, and next months. Notably, the moving average information is unavailable for the initial and final months based on this calculation approach.

In the fourth month, the anti-SARS-CoV-2 (N) showed their maximal mean value, exhibiting a 79% rise compared to the first month (cutoff index (COI) 78.87 ± 2.95 vs. 44.03 ± 2.85, *p* < 0.05). Over the following eight months, the peak value seen in the fourth month experienced a mild 28% fall, keeping a rather elevated level of 56.36 ± 3.29 by the end of the first year. Variations were seen in the following months, with values slightly above those of the first month Fig. 2C). To affirm our findings and provide a comprehensive visualization of the trends, we operated a moving average chart. This method helped smooth out short-term fluctuations and highlighted the underlying trend over time, substantiating the observed rise, peak, and subsequent decline in anti-SARS-CoV-2 (N) levels (Fig. 2D).Conversely, anti-SARS-CoV-2 (S) exhibited a consistent increase at each three-month interval over the 15 months, culminating in a statistically significant peak mean value at that time point(U/mL 212.44 ± 38.87 vs. 858.51 ± 123.33, *p* < 0.0001), after which the tendency to decline was observed (Fig. 2A). The SEM for the late months (16–21) is big due to small sample size.

To determine the statistical significance of these observations, paired t-test analyses were performed on patients having same-month results. The results showed significant development between the selected months. The paired differences mean values and 95% confidence intervals were as follows: between the third and first month, 74.07 (95% CI 3.72–144.41); between the sixth and first month, 112.18 (95% CI 29.14–195.22); between the ninth and first month, 197.04 (95% CI 32.64–361.45); and between the twelfth and sixth months, 233.3 (95% CI 81.87–384.76).

During the declining phase, anti-SARS-CoV-2 (N) became undetectable in the serum of 24 patients, while anti-SARS-CoV-2 (S) reached undetectable levels in one patient. Within this cohort of convalescent patients, a mere 5.6% (n = 90) did not produce determinate levels of anti-SARS-CoV-2 (N) and 6.5% (n = 105) did not manifest determinate levels of anti-SARS-CoV-2 (S).

### Association of antibody kinetic with disease severity and demographic parameters

The non-adjusted binary logistic regression showed that being seropositive for anti-SARS-CoV-2 (N) and (S) was statistically significantly associated with the patient’s age, gender, Rh factor, and severity of COVID-19 infection. In addition, anti-SARS-CoV-2 (N) might also be associated with comorbidities such as diabetes and oncological diseases (Table 2).

**Table 2:** Associations between potential seropositive predictors in COVID-19 convalescent patients for anti-SARS-CoV-2 (N) and (S) antibodies: univariate logistic regression.

Potential predictors	Serum anti-SARS-CoV-2 (N)				Serum anti-SARS-CoV-2 (S)			
	Crude OR	95% CI		*P* value	Crude OR	95% CI		*P* value
Age	0.021	1.015	1.029	< 0.001	0.022	1.016	1.030	< 0.001
Gender, female	1.634	1.306	2.044	< 0.001	1.539	1.227	1.930	< 0.001
BMI, overweight	0.947	0.749	1.198	0.652	1.056	0.834	1.337	0.653
Smoking	1.042	0.751	1.445	0.808	1.152	0.830	1.600	0.396
Rh ( +)	0.668	0.484	0.923	0.014	0.648	0.469	0.895	0.009
COVID-19 infection severity	1.350	1.159	1.572	< 0.001	1.320	1.134	1.536	< 0.001
Pneumonia	0.955	0.593	1.539	0.851	0.808	0.502	1.301	0.381
Diabetes	1.648	1.028	2.644	0.038	1.479	0.933	2.345	0.096
Oncological diseases	0.402	0.154	1.052	0.063	0.504	0.193	1.319	0.163

Female gender, Rh-negative blood type, and the severity of COVID-19 infection were shown to be substantially associated with the production of anti-SARS-CoV-2 (N) in the final adjusted multivariate logistic regression model that included all possible factors (Table 3). Specifically, those who had moderate or severe clinical symptoms of COVID-19 infection showed 71.6% higher chance to develop anti-SARS-CoV-2 (N) than those with asymptomatic or mild symptoms (odds ratio [OR] = 0.284, 95% confidence interval [CI]: 0.130–0.620, *p* = 0.002).

**Table 3:** Predictors of being seropositive in COVID-19 convalescent patients for anti-SARS-CoV-2 (N) and (S) antibodies: multivariate logistic regression.

Predictors	Anti-SARS-CoV-2 (N)				Anti-SARS-CoV-2 (S)			
	OR	95% CI		*P* value	OR	95% CI		*P* value
Severity, severe/moderate	0.284	0.130	0.620	0.002	0.266	0.128	0.552	< 0.001
Gender, female	0.363	0.197	0.669	0.001	0.554	0.312	0.984	0.044
Rh(-)	3.565	1.085	11.709	0.036	1.695	0.748	3.838	0.206

Similarly, females had 63.7% higher odds ratio of developing anti-SARS-CoV-2 (N) than males (OR = 0.363, 95% CI: 0.197–0.669, *p* < 0.001). Notably, people with Rh-negative blood type have approximately 3.565 times higher odds of developing anti-SARS-CoV-2 (N) than those with Rh-positive blood type (OR = 3.565, 95% CI: 1.085–11.709, *p* = 0.036).

The only significant indicators of anti-SARS-CoV-2 (S) were COVID-19 infection severity and gender. Individuals with moderate or severe COVID-19 infection had 73.4% higher chances of developing anti-SARS-CoV-2 (S) than those with asymptomatic or mild disease (OR = 0.266, 95% CI 0.128–0.552, *p* < 0.001). Furthermore, females were 44.6% more likely than males to produce anti-SARS-CoV-2 (S) (OR = 0.554, *p* = 0.044).

This study used linear mixed models (LMMs) to evaluate the connections between possible features (age, gender, disease severity, and rhesus factor) and the kinetics of anti-SARS-CoV-2 (N) and (S) antibodies (Table 4).

**Table 4:** Factors associated with the kinetics of anti-SARS-CoV-2 (N) and (S) antibodies for COVID-19 convalescent patients during 15 months of follow-up.

Factors	Anti-SARS-CoV-2 (N)				Anti-SARS-CoV-2 (S)			
	Antibodies peak		Antibodies decay		Antibodies peak		Antibodies decay	
	*P* value	95% CI	*P* value	95% CI	*P* value	95% CI	*P* value	95% CI
Age, > 48 years	< 0.001	(− 0.85; − 0.34)	0.007	(0.25; 1.63)	< 0.001	(− 55.01; − 23.38)	< 0.001	(67.96; 132.56)
Rh, (+)	0.001	(0.21; 0.78)	< 0.001	(− 2.53; − 1.09)	< 0.001	(20.65; 52.32)	0.002	(− 96.75; − 22.48)
Gender, female	0.662	(− 0.21; 0.33)	0.001	(0.47; 1.82)	< 0.001	(14.47; 45.60)	< 0.001	(103.99; 174.58)
Severity, severe/moderate	0.558	(− 0.33; 0.18)	0.213	(− 0.24; 1.06)	0.473	(− 9.79; 21.083)	< 0.001	(− 140.23; − 75.65)

Our goal was to uncover parameters that influence the rate of reaching antibody peak and later breakdown rates.

Factors predicting the kinetics for anti-SARS-CoV-2 (N) were the following:

- Peak: Antibodies in Rh-positive subjects (*p* = 0.001; 95% CI 0.21, 0.78) and those over 48 years old (*p* < 0.001; 95% CI − 0.85, − 0.34) peaked faster in comparison to Rh-negative subjects and those under 48 years old. Gender and illness severity had no significant influence on reaching a peak.
- Decay: Females (*p* = 0.001; 95% CI 0.47, 1.82), those under 48 years old (*p* = 0.007; 95% CI 0.25, 1.63), and Rh-negative subjects (*p* < 0.001; 95% CI − 2.53, − 1.09) had a quicker decline rate.

Factors predicting the kinetics for anti-SARS-CoV-2 (S) are the following:

- Peak: Rh-positive individuals (*p* < 0.001; 95% CI − 55.01, − 23.38), females (*p* < 0.001; 95% CI 14.47, 45.60), and those over 48 years old (*p* < 0.001; 95% CI − 55.01, − 23.38) exhibited higher rates of reaching antibody peak, with no significant influence from disease severity.
- Decay: Notably, all four factors were significantly associated with a faster decay rate for anti-SARS-CoV-2 (S): females (*p* < 0.001; 95% CI 103.99, 174.58), individuals under 48 years old (*p* < 0.001; 95% CI 67.96, 132.56), Rh-negative individuals (*p* = 0.002; 95% CI − 96.75, − 22.48), and individuals with asymptomatic or mild disease (*p* < 0.001; 95% CI − 140.23, − 75.65).

**Figure 3:**
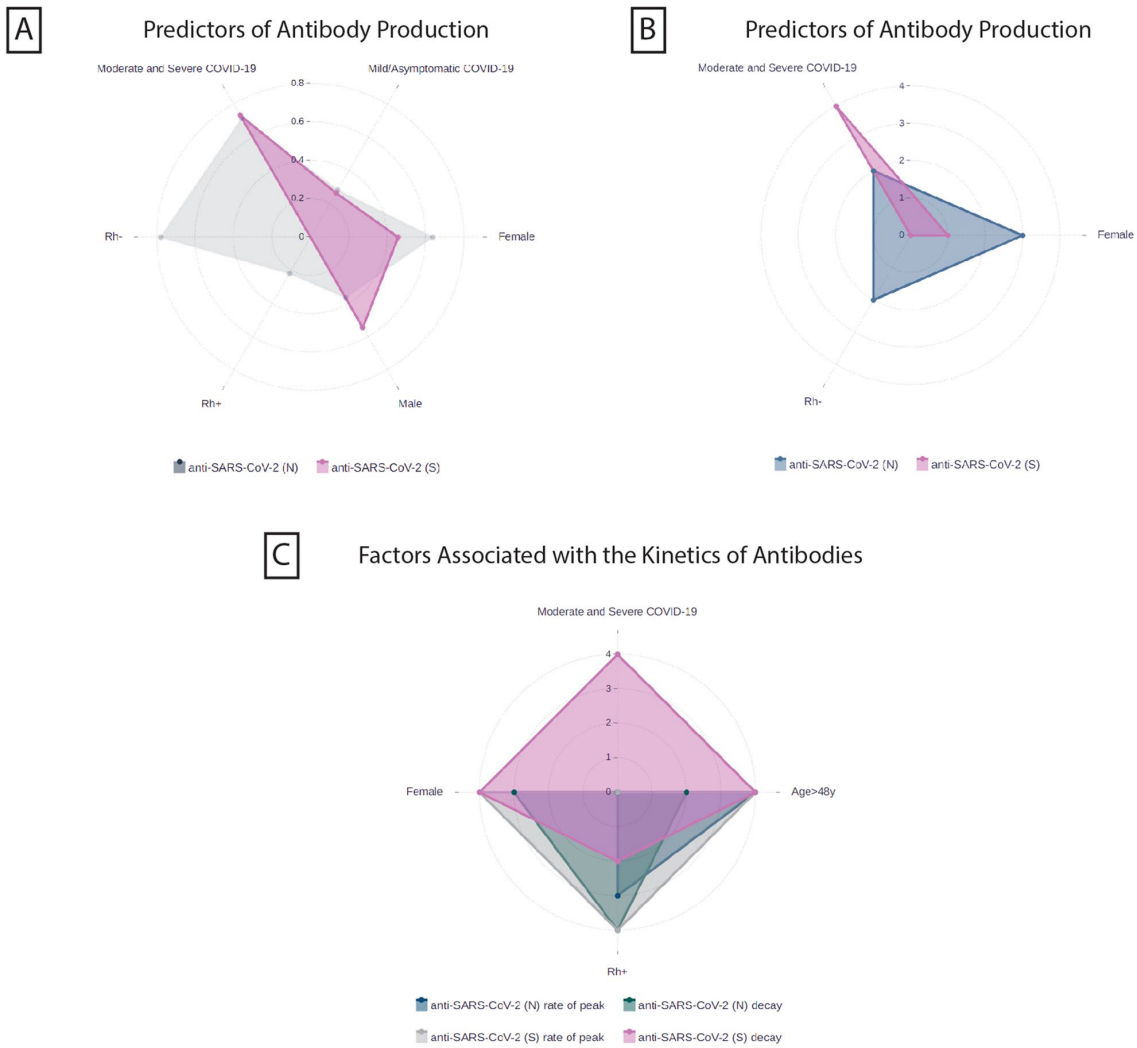
Factors associated with anti-SARS-CoV-2 antibody production and kinetics. The statistical analysis was performed by a final adjusted multivariate logistic regression model (**a**, **b**) and linear mixed models (**c**) and data are exhibited with odds ratios (**a**) and *p* values (**b**, **c**) on the graphs. (**A**)—Female gender, Rh-negative blood type, and the severity of COVID-19 infection are associated with the anti-SARS-CoV-2 (N) and (S) production. Odds ratios are demonstrated in different colored dots to show the power of association for each factor ranged from 0–0.8. (**B**)—On the graph b the dots are represented *p* values to demonstrate the probability of the mentioned factors association with antibody production. The *p* values are coded with the following numbers: *p* < 0.001 = 4, 0.001–0.002 = 3, 0.002–0.01 = 2, 0.01–0.05 = 1, > 0.05 = 0. (**C**)—Graph c demonstrates the factors linked with the rate of reaching antibody peak and further decay rates. The p values are coded with the following numbers: *p* < 0.001 = 4, 0.001–0.002 = 3, 0.002–0.01 = 2, 0.01–0.05 = 1, > 0.05 = 0.

In summary, the factors linked to the production of anti-SARS-CoV-2 antibodies and their impact on the rate of achieving antibody peak levels, as well as following decline rates, are outlined on graph (Fig. 3A-C).

**Figure 4:**
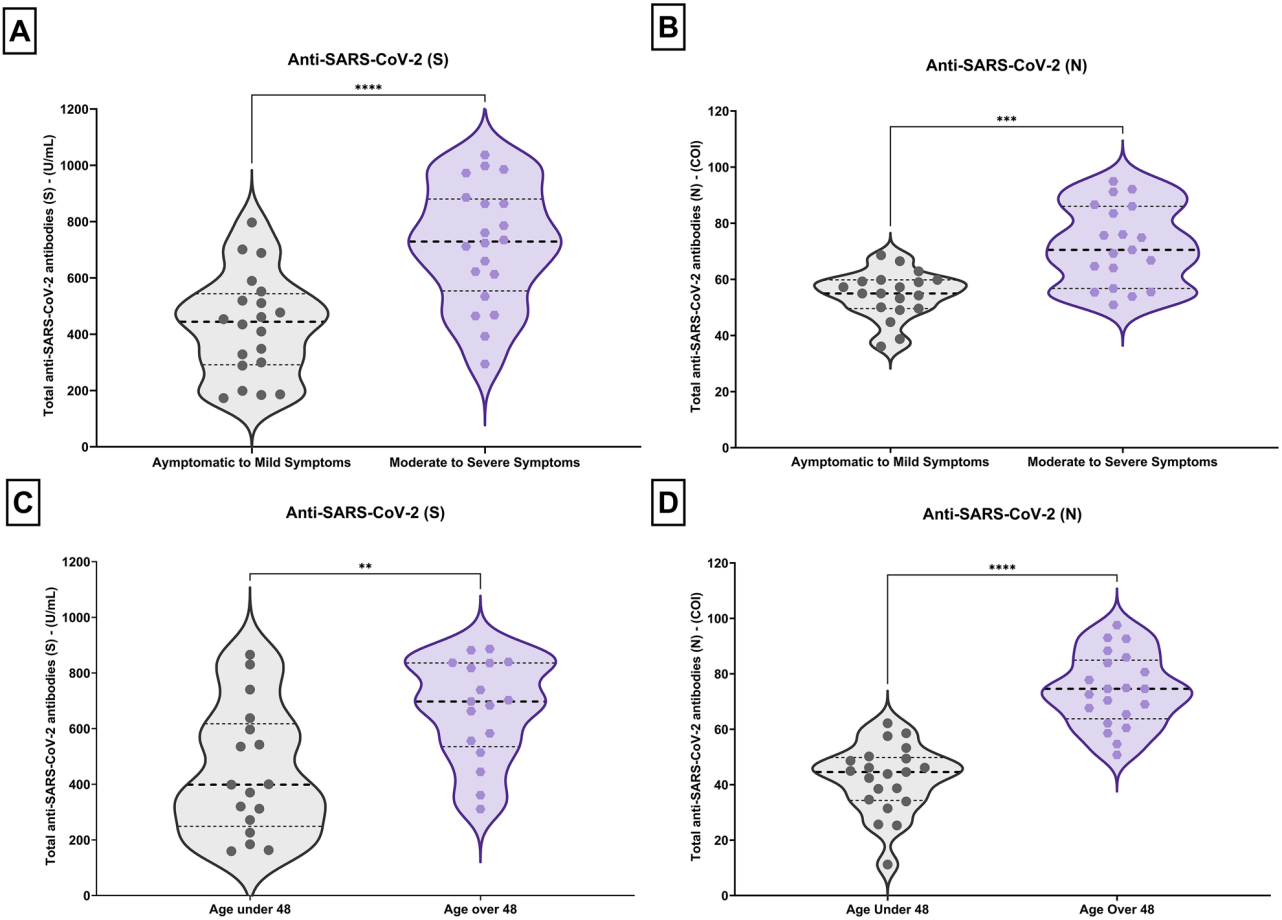
Violin plots for the distribution of antis-SARS-CoV-2 (S) & (N) for severity (**A**, **B**) and age (**C**, **D**) groups. Wider area of the violin plot represents a higher distribution of antibodies’ titer, and the thinner area corresponds to a lower distribution. Dots denote means for each month. Horizontal black dotted lines show the median of the antibodies’ titer and horizontal black dot lines represent the interquartile range (IQR) of the titer.

Besides, the distribution of anti-SARS-CoV-2 (N) and (S) antibody levels according to disease severity and age was vividly portrayed using violin plots (Fig. 4). These plots offer a comprehensive view of antibody levels' spread and central tendencies within different age groups and severity groups (moderate to severe and asymptomatic to mild).

A thorough analysis of data reveals a particularly important observation: antibody levels in the 3rd quartile (75th percentile) of the asymptomatic to mild group are much lower than the median antibody level observed in the moderate to severe group for both anti-SARS-CoV-2 (N) and (S) (Fig. 4A, B). The same pattern can be visualized in the patients < 48 years of age in contrast to older patients (> 48 years).

The violin plots showing the distribution of anti-SARS-CoV-2 (S) and anti-SARS-CoV-2 (N) proteins in the mild group provide a slight view of the variation in immune response to different viral components. Figure 4 presents violin plots delineating the analysis of antibody levels utilizing the Mann–Whitney U test. The key findings are as follows: A comparative assessment between anti-SARS-CoV-2 (S) and anti-SARS-CoV-2 (N) antibody titers, respectively, in patients displaying asymptomatic to mild symptoms versus those manifesting moderate to severe symptoms revealed a statistically significant distinction (*p* < 0.0001)(Fig. 4A, B).

The scattering of data points for anti-SARS-CoV-2 (S) is exhibited, as compared to the more concentrated and compact distribution for anti-SARS-CoV-2 (N).

Examining the violin plots of patients based on age indicates a considerable concentration of younger subjects exhibiting lower antibody levels, differing from the older age group (Fig. 4C, D).

A comparative assessment between anti-SARS-CoV-2 (S) and anti-SARS-CoV-2 (N) antibody titers, respectively, across two age groups (below 48 years and above 48 years) also demonstrated a statistically significant discrepancy (*p* < 0.0001) (Fig. 4C, D).

## Discussion

In this population-based study, we conducted an extensive evaluation of the long-term humoral immune response, specifically focusing on the dynamics of anti-SARS-CoV-2 (N) and (S) antibodies, over 21 months following COVID-19 infection.

Based on a thorough literature review, this investigation excels in being one of the most extensive and enduring, by virtue of its substantial sample size and duration in analyzing the dynamics of long-term humoral immunity after COVID-19 infection.

This study demonstrates marked seropositivity rates for both anti-SARS-CoV-2 (N) and (S) antibodies over a comprehensive period of evaluation. Around 94.5% of participants showed positive values for anti-SARS-CoV-2 (N), while 95.9% demonstrated positivity for anti-SARS-CoV-2 (S) antibodies, reflecting a sustained antibody-mediated humoral immune response among the cohort during the 21 months of measurement.

A small proportion of convalescent patients (5.6% for anti-SARS-CoV2 (N) and 6.5% for anti-SARS-CoV2 S) did not produce detectable levels of antibodies against SARS-CoV-2. This phenomenon of non-response highlights the variability in individual immune reactions and emphasizes the need for a comprehensive understanding of immune factors influencing antibody production.

Individuality in the immune response shined through as we observed the puzzling presence of anti-SARS-CoV-2 (S) without detectable anti-SARS-CoV-2 (N) in 1% of our subjects, which submitted the need for dissecting the diverse factors shaping the response and exploring their potential impact.

In the study conducted by Rosati et al.^30^ and her colleagues, the presented outcomes of a sequential analysis of anti-SARS-CoV-2 antibodies among convalescent patients during the 14-month median follow-up period after the initial onset of symptoms reveals the enduring presence of both antibodies. Notably, the study highlights that anti-SARS-CoV-2 (S) exhibited greater persistence compared to anti-SARS-CoV-2 (N) which corresponds to our data Rosati et al.^31^.

Another investigation led by Mar Masiá and collaborators in 2021, focusing exclusively on hospitalized patients, revealed that a considerable proportion of individuals hospitalized with COVID-19 INFECTION retained detectable levels of anti-SARS-CoV-2 (S) even one year post the initial acute infection. Our investigation found a unique trend for anti-SARS-CoV-2 (N), in contrast to the findings of Masiá et al., who reported a drop in anti-SARS-CoV-2 (N) levels in over 50% of hospitalized patients Masiá et al.^32^. In our study there was a noticeable surge between months 4 and 6, followed by a hesitant, steady drop. Remarkably, anti-SARS-CoV-2 (N) levels remained significantly elevated throughout the 21-month follow-up, with only 24 of the total participants forfeiting detectable levels. This implies that the initial immune response to the nucleocapsid protein is very persistent and continues to function to some extent even in the long term.

Contrastively, anti-SARS-CoV-2 (S) antibodies did not follow suit to anti-SARS-CoV-2 (N) antibodies but sustained a progressive ascent throughout the 15-month follow-up period, this climb ascended to a significant static peak at the study’s conclusion, yielding a long lasting and plausible immune response against their specific target protein.

Collectively, these observations provide insights into the temporal dynamics of the humoral immune response to SARS-CoV-2 virus. On the other hand, the constant elevation of anti-SARS-CoV-2 (S) antibody levels highlights the establishment of a durable immune response against the spike protein. These findings contribute to our understanding of the complexities of COVID-19 long-term humoral immune response and can have implications for vaccine development and immune monitoring strategies.

The literature review highlights a scarcity of comprehensive, extended-duration studies for assessing the longevity of humoral immune responses in both convalescent and vaccinated patients. Our study, by virtue of its design and scope, holds the potential to serve as a foundational database for facilitating such comparative analyses in the future.

However, it's important to note that this study provides an observational correlation rather than a causal relationship. The factors influencing these antibody responses could be influenced by a wide array of variables, including individual immune profiles, health-related and demographic conditions.

Therefore, further research is needed to unravel the underlying mechanisms driving this correlation and to ascertain whether it holds true across different populations, timeframes, and disease contexts.

The subsequent objective of our study is supposed to identify the specific predictors contributing to the development of antibodies.

In this content we can separate two types of predictors: general predictors shared by both anti-SARS-CoV-2 (N) and anti-SARS-CoV-2 (S) and specific to anti-SARS-CoV-2 (N).

We found that disease severity and female gender served as general predictors shared by both anti-SARS-CoV-2 (N) and anti-SARS-CoV-2 (S). In fact, individuals with more severe COVID-19 infection cases showed a higher likelihood of developing both antibodies. At the same time women displayed a higher likelihood of developing both types of antibodies compared to men.

Analyzing the role of the Rh factor in the production of anti-SARS-CoV-2 antibodies, we found that only Rh-negative individuals demonstrated a higher probability of anti-SARS-CoV-2 (N) production, but not anti-SARS-CoV-2 (S) antibody. Thus, we settled Rh factor as the specific predictor of anti-SARS-CoV-2 (N).

Beyond mere quantification, our study delves deeper, dissecting the factors orchestrating the rise and fall of anti-SARS-CoV-2 (S) and anti-SARS-CoV-2 (N). Summarizing, we can assume that Rh-positive individuals and those over 48 years old generally displayed higher rates of reaching a peak for both anti-SARS-CoV-2 (N) and (S). Gender and age had a greater impact on the decay rates of both antibody types, with females and younger individuals expressing faster decay. We can highlight female gender as a specific predictor for the higher rate of reaching a peak of anti-SARS-CoV-2 (S), and disease severity—for anti-SARS-CoV-2 (S) decay rate, with asymptomatic or mild patients showing faster decay (Fig. 3).

However, the relationship between antibody levels and disease severity is not always straightforward. Some studies suggest that individuals with severe COVID-19 infection cases often exhibit elevated antibody levels, indicative of an enhanced immune response, this activity does not guarantee beneficial outcomes^42,43^, as it may trigger an excessive inflammatory reaction known as a cytokine storm, causing tissue damage and exacerbated clinical symptoms. Our data similarly reveals a statistically significant high titer of antibodies in the moderate and severe patient groups congruently with those studies.

The intricate relationship between age, humoral immune responses, and disease severity in the context of COVID-19 infection has garnered substantial attention in the realm of research. Age, as an established determinant of immune system function, plays a pivotal role in shaping immune responses and susceptibility to infections^33,34^. It is a known fact that as individuals advance in age, their immune response undergoes significant changes, a phenomenon widely recognized as immunosenescence^35–38^. This phenomenon contributes to a gradual reduction in the efficacy of both innate and adaptive immune responses, encompassing the production of antibodies^39^. This decline can compromise the defense against infections and weaken the responsiveness to vaccinations.

Despite robust evidence that asserted a decline in humoral immune response influenced by age^40^, our investigation into anti-SARS-CoV-2 antibody titers unveils a contrasting perspective. Based on the 21-month follow-up of the 1611 participants we revealed elevated levels of anti-SARS-CoV-2 (S) and anti-SARS-CoV-2 (N) in older individuals (≥ 48 years) compared to their younger counterparts (< 48 years). These results were exhibited on violin plots where we can see the concentration of the majority of patients aged > 48 within the high antibody levels in all quartiles for both types of antibodies, more expressed in anti-SARS-CoV-2 (N). This finding indicates potential differences in immunological dynamics and points out the importance of understanding the antibody response in various age groups. Notably, this observation was confirmed by our earlier study^41^.

The antibody response patterns influenced by age-specific discrepancy and their potential downside deem the topic of immune response dynamics rather fascinating.

Amidst these considerations, age stands out as a critical factor influencing the risk of severe COVID-19 infection. Older individuals remain at a heightened risk of experiencing severe disease outcomes. Collectively, these findings underscore the intricate interplay between age, humoral immune responses, and the severity of disease in the COVID-19 infection context. The age-related decline in immune function, coupled with the complex dynamics between immune responses and disease outcomes, highlights the necessity for tailored approaches to vaccination and treatment, particularly among older individuals who may display diminished immune responsiveness and heightened vulnerability to severe disease manifestations.

Our observation of the association between the aging process and heightened humoral immune responses prompts the hypothesis of a compensatory mechanism aimed at counteracting reduced immune responsiveness which needs further confirmation with more thorough investigations, especially neutralizing function of the antibodies. This intriguing insight gains significance against the backdrop of well-documented declines in immune function with age. This avenue of inquiry beckons further exploration, aiming to unravel the intricate mechanisms underlying this compensatory phenomenon. Such exploration holds promise for a deeper comprehension of immune adaptations among older individuals, potentially leading to targeted interventions aimed at bolstering immunity within this vulnerable population.

With all positive results it is noteworthy to mention that one of the disadvantages of our study was the lack of clinical data on concomitant diseases, since this information was provided by the patients themselves. Although participants who reported receiving a second vaccination against SARS-CoV-2 or re-infection were excluded from our study, it is possible that some subjects continued participation in the study while being asymptomatic during the reinfection or keeping their vaccination status confidential.

## Conclusion

In conclusion, our investigation provides compelling evidence of sustained seropositivity rates for both anti-SARS-CoV-2 (N) and (S) among convalescent COVID-19 patients over a comprehensive 21-month evaluation period. Our findings also display distinct dynamics in the long-term antibody responses, with anti-SARS-CoV-2 (N) levels displaying remarkable persistence and anti-SARS-CoV-2 (S) antibodies exhibiting a progressive incline. Furthermore, our study identifies demographic and clinical characteristics, such as Rh status, age, gender, and disease severity, as significant predictors of antibody production and kinetics. These insights contribute to a deeper understanding of the factors influencing humoral immune responses against the SARS-CoV-2 virus, announcing future research endeavors and guiding the development of targeted interventions and vaccination strategies.

## Methods

### Participants and Samples

infectionThe primary aim of this prospective population-based study was to investigate dynamics of anti-SARS-CoV-2 (N) and (S) in long-term immunity during 21 months in 1611 convalescent patients across diverse regions of Armenia and glimpse factors influencing that. Commencing in August 2020 and concluding in March 2022, the enrollment period necessitated the fulfillment of stringent inclusion criteria. Participants were required to exhibit recent SARS-CoV-2 infection, validated through positive RT-PCR results (at least 3–4 weeks post initial positive testing), coupled with an absence of clinical symptoms during sample collection. Mandatory submission of official laboratory documentation corroborating the positive PCR test result underscored the rigor of participant selection. Exclusions comprised pregnant women, individuals under 18 years old, and those with primary or secondary immunodeficiency. A brief questionnaire concerning the current clinical symptoms and vaccination status of each participant was completed during each visit. During the sampling phase, vaccinated patients and patients with “COVID-19 infection” like symptoms were dropped out. As the dropout rate during the last months was very high, the study was terminated earlier than was planned.

This study measured the levels of anti-SARS-CoV-2 (N) and (S) in convalescent patients after 3–4 weeks of positive PCR testing, and then monthly collections extending over a span of 21 months. A meticulous tally of 6115 serum samples was systematically amassed from the cohort of 1611 convalescent patients at varying post-infection intervals. Within the data set of 6115 samples, 769 were acquired within a span of 365 days, whereas 286 samples underwent examination over an extended time frame of 450 days. In this study the monthly number of the subjects exceeds the sample size for a population-based survey in Armenia (384) in the period from 2 to 10 months (Supplementary Figure S2).

Additionally, the study encompassed control groups representing both pre-pandemic and pandemic groups, with only a single sampling event for each. The “pre-pandemic control group” incorporated serum samples from 71 healthy donors predating the SARS-CoV-2 pandemic period (spanning from 2017 to February 2020) and 100 healthy donors during the pandemic's early phases (from March 2020 to May 2020).

### Clinical measures

Within the framework of this study, a comprehensive questionnaire devised by the research team, comprising social-demographic, epidemiological, and clinical dimensions, facilitated the collection of pertinent data. After analyzing clinical symptoms, patients were categorized into four groups based on disease severity, using modified definitions from the WHO^44^. Classification encompassed asymptomatic individuals with positive PCR testing, mild cases meeting the COVID-19 INFECTION case definition lacking evidence of viral pneumonia or hypoxia, moderate cases exhibiting clinical signs of pneumonia with SpO2 ≥ 90% on room air, and severe cases with SpO2 < 90%.

### Laboratory measures

The study participants underwent a conventional venipuncture procedure at the Heratsi University Hospital Laboratory to collect their blood samples. Subsequent centrifugation segregated serum from blood elements, with the serum samples subjected to analysis for anti-SARS-CoV-2 (N) and (S) total antibodies, including IgG using a commercially available “Elecsys Anti-SARS-CoV-2” and “Elecsys Anti-SARS-CoV-2 S” assay from Roche Diagnostics.

The Elecsys Anti-SARS-CoV-2 assay is a qualitative test and the results were determined by a cutoff index (COI), where values of COI < 1 as negative and ≥ 1 as positive for anti-SARS-CoV-2 (N).

Conversely, the quantitative Elecsys Anti-SARS-CoV-2 S assay interpreted values below 0.8 U/ml as negative and values greater than or equal to 0.8 U/ml as positive. The Elecsys Anti-SARS-CoV-2 S is an immunoassay in vitro qualitative test used to detect human antibodies to SARS-CoV-2 S protein RBD in serum and plasma. The assay uses a recombinant RBD protein in a double-antigen sandwich assay, determining the qualitative antibody’s high affinity against SARS-CoV-2 S. The system’s linearity is limited to 250 U/mL, in our study we considered following SARS-CoV-2 infection, requiring the measurement of antibody levels beyond the Roche Elecsys system’s standard span. To extend the measurement range of the Roche Elecsys system, we took samples with anti-SARS-CoV-2 S concentrations and it was measured by dilution technique (Diluent Universal or Diluent Universal 2). The recommended dilution according to the FDA is 1:10 up to 1:100^47^. Serum with high antibody concentration samples were diluted (at least 250 uL) properly to bring them within the linear range of the assay. In addition, a rigorous calibration method was conducted to ensure accuracy and reliability across the extended measurement span. The remaining volume of more than 250 uL can be re-used if secure and stored immediately (2–8 C). Our modified assay protocol qualified the accurate measurement of SARS-CoV-2 antibody levels up to 2000 U/mL using the Roche Elecsys system.

### Ethical approval

The samples were handled according to standard operating procedures and the study was conducted following the principles of the Declaration of Helsinki and approved by the Ethics Committee (N 8–2/20; 02.07.2020 and 3-2/2020; 27.11.2020) of Yerevan State Medical University, with informed consent obtained from all participants.

### Statistical analyses

We used IBM^®^ SPSS^®^ v.22.0.0 and Stata/MP 15 statistical packages for our analyses to assess the distribution of socio–demographic and clinical characteristics of COVID-19 infection convalescent patients, using frequency and proportion for categorical variables, and mean and standard deviation (SD) for continuous variables. As a baseline measurement point, we used the date of the positive PCR test and reported the anti-SARS-CoV-2 (N) and (S) level measures kinetic over the following 15 months. We first tested the associations between each independent variable and the positive serology (titer ≥ 1.0) that showed statistical significance or close to statistical significance through univariate logistic regression and then derived a multivariate model for odds-ratio estimates to adjust for potential confounders and to explore potential effect modifications. Individuals reported ≥ 3 antibody results in a total number of measurements used for trends analysis. For the anti-SARS-Cov-2 antibody levels time-repeated measures, we applied a linear mixed effect model to estimate the rate of antibody responses from the maximum observed result and their association with age, gender, severity, and Rh (+) factor^45,46^. All results were derived from 2-tailed tests and reported with *p* value level of statistical significance < 0.05.

### Supplementary Information

Supplementary Information 1.

Supplementary Information 2.

Supplementary Information 3.

## Data Availability

Data can be made available by the corresponding author upon reasonable request.
