# Author Correction: Tracking the evolution of anti-SARS-CoV-2 antibodies and long-term humoral immunity within 2 years after COVID-19 infection

**DOI:** 10.1038/s41598-024-70482-8

**Published:** 2024-08-22

**Authors:** Mariam Movsisyan, Nune Truzyan, Irina Kasparova, Armine Chopikyan, Ra’ed Sawaqed, Alexandra Bedross, Meline Sukiasyan, Karen Dilbaryan, Sanobar Shariff, Burhan Kantawala, Gohar Hakobjanyan, Gayane Petrosyan, Armine Hakobyan, Konstantin Yenkoyan

**Affiliations:** 1 Department of Allergology and Clinical Immunology, Yerevan State Medical University Named After Mkhitar Heratsi, Yerevan, Armenia; 2 Cobrain Center, Yerevan State Medical University Named After Mkhitar Heratsi, Yerevan, Armenia; 3 Department of Histology, Yerevan State Medical University Named After Mkhitar Heratsi, Yerevan, Armenia; 4 Department of Public Health and Healthcare Organization, Yerevan State Medical University Named After Mkhitar Heratsi, Yerevan, Armenia; 5 General Medicine Faculty, Yerevan State Medical University Named After Mkhitar Heratsi, Yerevan, Armenia; 6 Laboratory‑Diagnostic Center of Heratsi Clinical Hospital, Yerevan State Medical University Named After Mkhitar Heratsi, Yerevan, Armenia; 7 Neuroscience Laboratory, Cobrain Center, Yerevan State Medical University Named After Mkhitar Heratsi, 0025 Yerevan, Armenia; 8 Department of Biochemistry, Yerevan State Medical University Named After Mkhitar Heratsi, Yerevan, Armenia

Correction to: *Scientific Reports* 10.1038/s41598-024-64414-9, published online 11 June 2024

The original version of this Article contained an error in the spelling of the author Burhan Kantawala which was incorrectly given as Burhan Kwantala.

The original Article has been corrected.

