# Supplementary material for: Tracking the evolution of anti-SARS-CoV-2 antibodies and long-term humoral immunity within 2 years after COVID-19 infection
Source: Sci Rep. 2024 Jun 11;14:13417. doi: 10.1038/s41598-024-64414-9 (PMC11167004; doi:10.1038/s41598-024-64414-9)
Supplement: Supplementary file 1 — Supplementary Information 1. [file 41598_2024_64414_MOESM1_ESM.pdf]

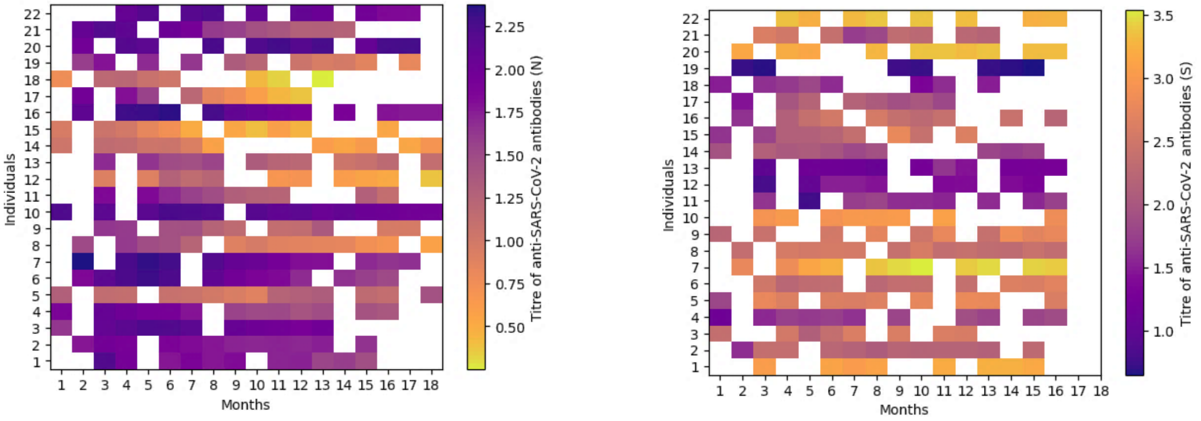


**Supplementary Figure S1.** Time-related changes of anti-SARS-CoV-2 (N)(a) and anti-SARS-CoV-2 (S)(b) over 18 months, displaying the dynamic variations of 22 individuals throughout the follow-up period. Titer of antibodies was calculated using log10 scale.
