# Supplementary figures and images for: Tracking the evolution of anti-SARS-CoV-2 antibodies and long-term humoral immunity within 2 years after COVID-19 infection
Source: Sci Rep. 2024 Jun 11;14:13417. doi: 10.1038/s41598-024-64414-9 (PMC11167004; doi:10.1038/s41598-024-64414-9)

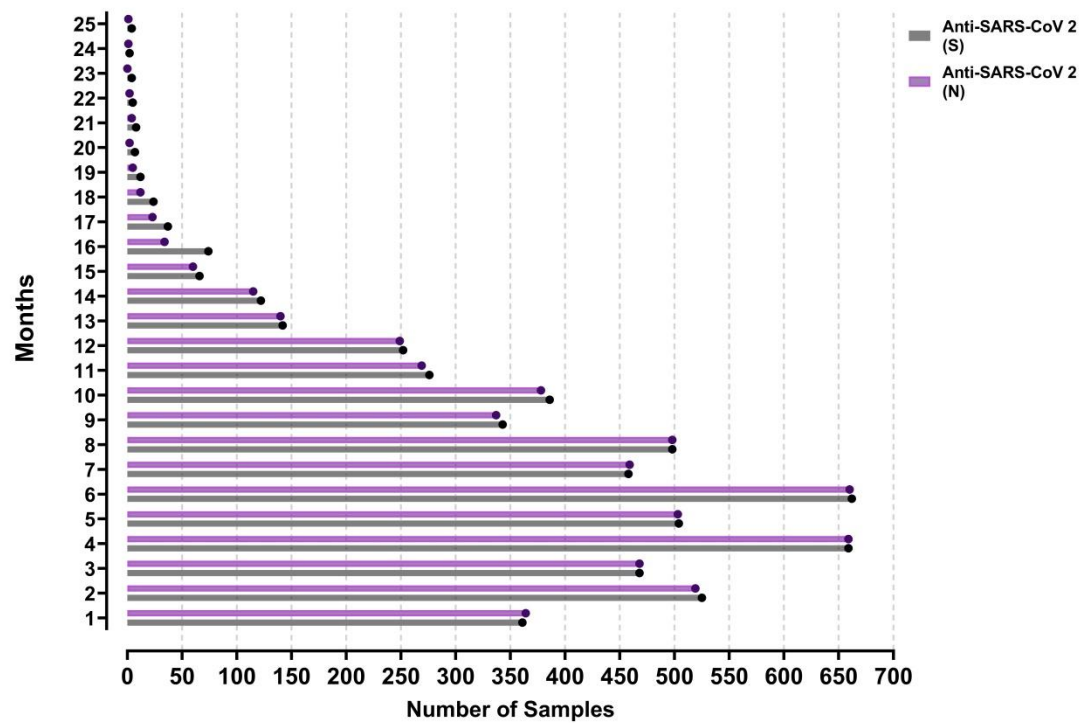

**Supplementary Figure S2.** The subjects involved in the study during 21 months follow up.

Supplement: Supplementary file 2 — Supplementary Information 2. [file 41598_2024_64414_MOESM2_ESM.pdf]
