# Supplementary material for: Tracking the evolution of anti-SARS-CoV-2 antibodies and long-term humoral immunity within 2 years after COVID-19 infection
Source: Sci Rep. 2024 Jun 11;14:13417. doi: 10.1038/s41598-024-64414-9 (PMC11167004; doi:10.1038/s41598-024-64414-9)
Supplement: Supplementary file 3 — Supplementary Information 3. [file 41598_2024_64414_MOESM3_ESM.pdf]

Supplementary Table S1. The mean time between samplings and mean number of samples per individuals during study.

	**Total**		**Asymptomatic+Mild**		**Moderate+Severe**		**P value**
	Mean	Standard Error of Mean	Mean	Standard Error of Mean	Mean	Standard Error of Mean	
**Time between samplings**	52.43	1.35	52.85	1.82	51.84	2.01	>0.05
**Number of samplings**	3.68	0.08	3.54	0.09	3.92	0.13	<0.05
